# Siaα2-3Galβ1- Receptor Genetic Variants Are Associated with *Influenza A(H1N1)pdm09* Severity

**DOI:** 10.1371/journal.pone.0139681

**Published:** 2015-10-05

**Authors:** Alvino Maestri, Vinicius Albuquerque Sortica, Luciana Tovo-Rodrigues, Mirleide Cordeiro Santos, Luana Barbagelata, Milene Raiol Moraes, Wyller Alencar de Mello, Leonor Gusmão, Rita Catarina Medeiros Sousa, Sidney Emanuel Batista dos Santos

**Affiliations:** 1 Laboratory of Human and Medical Genetics, Federal University of Pará, Belém, Pará, Brazil; 2 Center of Cancer Studies, Federal University of Pará, Belém, Pará, Brazil; 3 Laboratory of Respiratory Viruses, Virology Section, Evandro Chagas Institute, Ananindeua, Pará, Brazil; 4 Institute of Molecular Pathology and Immunology, University of Porto (IPATIMUP), Porto, Portugal; 5 Tropical Medicine Institute, Federal University of Pará, Belém, Pará, Brazil; 6 Centre of Epidemiological research, Federal University of Pelotas, Pelotas, Rio Grande do Sul, Brazil; Alberta Provincial Laboratory for Public Health/ University of Alberta, CANADA

## Abstract

Different host genetic variants may be related to the virulence and transmissibility of pandemic *Influenza A(H1N1)pdm09*, influencing events such as binding of the virus to the entry receptor on the cell of infected individuals and the host immune response. In the present study, two genetic variants of the *ST3GAL1* gene, which encodes the Siaα2-3Galβ1- receptor to which *influenza A(H1N1)pdm09 virus* binds for entry into the host cell, were investigated in an admixed Brazilian population. First, the six exons encoding the *ST3GAL1* gene were sequenced in 68 patients infected with strain A(H1N1)pdm09. In a second phase of the study, the rs113350588 and rs1048479 polymorphisms identified in this sample were genotyped in a sample of 356 subjects from the northern and northeastern regions of Brazil with a diagnosis of pandemic influenza. Functional analysis of the polymorphisms was performed *in silico* and the influence of these variants on the severity of infection was evaluated. The results suggest that rs113350588 and rs1048479 may alter the function of ST3GAL1 either directly through splicing regulation alteration and/or indirectly through LD with SNP with regulatory function. In the study the rs113350588 and rs1048479 polymorphisms were in linkage disequilibrium in the population studied (D’ = 0.65). The GC haplotype was associated with an increased risk of death in subjects with influenza (OR = 4.632, 95% CI = 2.10;1.21). The AT haplotype was associated with an increased risk of severe disease and death (OR = 1.993, 95% CI = 1.09;3.61 and OR 4.476, 95% CI = 2.37;8.44, respectively). This study demonstrated for the first time the association of *ST3GAL1* gene haplotypes on the risk of more severe disease and death in patients infected with *Influenza A(H1N1)pdm09 virus*.

## Introduction

Sialic acids are compounds derived from neuraminic acid which belong to a large family of complex nine-carbon sugars usually bound to other carbohydrates through α-ketosidic bonds. In mammals, sialic acids are found at the non-reducing end of glycoconjugates [1]. Influenza viruses are the oldest and most important examples of viruses that recognize sialic acids as the surface receptor for entry into host cells and their binding and propagation through interaction with these receptors have been well documented [2].

Avian flu virus strains preferentially bind to sialic acids linked to galactose through an α2–3 bond, while human flu virus strains preferentially attach to sialic acids linked to galactose through an α2–6 bond [3,4]. In contrast to other human viruses, the *Influenza A(H1N1)pdm09 virus* showed strong tropism for the two types of receptors during the 2009 pandemic [5,6]. This feature may explain the pandemic potential acquired by this virus, since it permitted the virus of swine origin to bind to Siaα2-6Gal (NAC) receptors of the upper respiratory tract, facilitating interpersonal transmission. On the other hand, maintenance of the capacity to bind to Siaα2-3Galβ1- permitted the virus to replicate in the lower respiratory tract, a fact explaining more severe cases of influenza such as severe viral pneumonias observed even in young adults without comorbidities [3,4,6].

Different host genetic variants may be related to the virulence and transmissibility of pandemic *Influenza A(H1N1)pdm09*, influencing events such as binding of the virus to the entry receptor on the cell of infected individuals and the host immune response [7]. The *ST3GAL1* gene (ST3 beta-galactosidase alpha–2,3-sialyltransferase 1) is located on the long arm of chromosome 8 (8q24.22) and encodes the Siaα2-3Galβ1- receptor. Different polymorphisms have been described in this gene. Three SNPs (rs939024, rs2978041 and rs2945733) have so far been identified in codifying regions related to bipolar disorders, but not to infectious diseases in humans [8,9,10].

*ST3GAL1* gene variants may be related to a higher or lower expression of the receptor on the surface of pneumocytes and thus interfere with the capacity of infection of the *Influenza A(H1N1)pdm09 virus* in cells of the lower respiratory tract [5,6], contributing to complications of this disease. Therefore, the present study investigated genetic variants of the *ST3GAL1* gene and correlated the finding with the progression of *Influenza A(H1N1)pdm09* infection in a Brazilian population.

## Results

### Clinical and demographic features

The demographic and clinical features of the participants are shown in Table 1, in which the 356 patients were divided into three groups according to severity: one of group of patients with classical symptoms who did not require hospitalization (n = 157), one group with severe acute respiratory syndrome (SARS) requiring hospitalization and survived to infection (n = 123), and a group of patients who was hospitalized but died due to infection (n = 76). There was a predominance of women in all groups (58%, 62.6% and 69.7% of non-hospitalized patients, hospitalized patients and patients who died, respectively). Patients who died were older when compared to the other two groups (p < 0,001). Among the comorbidities observed, metabolic disorders (p < 0.001), immunosuppression (p < 0.001) and obesity (p = 0.001) were associated with more severe disease, as was an abnormal chest X-ray (p < 0.001). The frequency of pregnancy, smoking, obesity, lung disease, heart disease, nephropathy or hemoglobinopathies did not differ significantly between groups. However, the absence of comorbidities was a protective factor in the sample (p = 0.049); in this respect, 72% of the subjects who did not require hospitalization had no associated diseases.

**Table 1:** Clinical and demographic characteristics of the patients infected with *Influenza A(H1N1)pdm09*. Age is presented as range (mean ±SD), all other variables are presented as number (%).

Characteristics	Not hospitalized	Hospitalized		P
		Survived	Died	
N	157	123	76	
Gender M/F	68/91 (58.0 F)	46/77 (62.6 F)	23/53 (69.7 F)	0.219
Age Range	1–66 (23.2 ± 13.6)	1–71 (21.4 ± 14.9)	1–80 (29.8 ± 15.8)	<0.001
Pregnant*	20 (29.0)	16 (29.1)	16 (39.0)	0.490
Smoking	6 (3.8)	6 (4.9)	7 (9.2)	0.257
Abnormal chest radiograph	0 (0)	59 (48.0)	48 (63.2)	<0.001
Without comorbidities	113 (72.0)	77 (62.6)	43 (56.6)	0.049
Chronic lung disorder	28 (17.8)	33 (26.8)	11 (14.5)	0.066
Chronic cardiovascular condition	10 (6.4)	7 (5.7)	8 (10.5)	0.393
Metabolic disorder	2 (1.3)	3 (2.4)	10 (13.2)	<0.001
Immunosuppression	0 (0)	1 (0.8)	6 (7.9)	<0.001
Obesity	1 (0.6)	1 (0.8)	6 (7.9)	0.001
Hemoglobinopathy	1 (0.6)	1 (0.8)	1 (1.3)	0.867
Chronic kidney disease	1 (0.6)	2 (1.6)	1 (1.3)	0.726

*Pregnant: Pregnant women among women of childbearing age (15–49 years)

### Population structure

The mean genetic contributions of the parental groups forming the population studied are shown in Table 2. Significant differences in European and African genetic contributions were observed between groups (p = 0.004 and p = 0.007, respectively). There was a higher European genetic contribution among non-hospitalized patients and a higher African genetic contribution among patients who died.

**Table 2:** Mean and range values for the genetic ancestry proportion (%) of the patients infected with *Influenza A(H1N1)pdm09*. Genetic ancestry proportions (%) are presented as mean ± SD (minimum—maximum)

Genetic Ancestry	Not hospitalized		Hospitalized				P
			Survived		Died		
Native American	23.6 ± 1.4	(2.9–89.7)	25.9 ± 1.5	(4.1–82.1)	28.2 ± 2.3	(4.5–90.6)	0.178
European	61.4 ± 1.6	(6.7–91.9)	56.4 ± 1.6	(9.5–89.2)	53.5 ± 2.4	(5.9–93.0)	0.004
African	14.9 ± 0.8	(2.7–61.0)	17.7 ± 1.0	(3.2–67.6)	18.3 ± 1.3	(2.4–50.4)	0.007

### Genotyping features

The allele and genotype frequencies of the *ST3GAL1* gene polymorphisms did not deviate from Hardy-Weinberg equilibrium. The polymorphisms rs113350588 located in exon four and rs1048479 located in exon eight result in synonymous substitutions of an aspartate (D) at position 95 and of a serine (S) at position 273 of the protein, respectively. *In silico* functional analysis suggests that both variants may have putative direct and indirect effect on gene regulation (Table A in S1 File). Splicing analyses suggested that both SNPs may alter splicing of the transcript and consequently the isoforms of the protein. The substitution of guanine (G) for adenine (A) in rs113350588 promotes a change in an exonic splicing enhancer site (disrupting the sites for SF2/ASF proteins interactions), while the substitution of cytosine (C) for thymine (T) in rs1048479 activates a cryptic acceptor site, with the presence of one or more cryptic branch points. These polymorphisms were in linkage disequilibrium (D’ = 0.65) in the population studied, resulting in four haplotype alleles that form nine observable genotypes (diplotypes) (Tables B and C in S1 File).

Moreover, analyses conducted with HaploReg 3.0 showed that rs1048479 is in linkage disequilibrium with two other polymorphisms (rs2142306 and rs276865) in all populations deposited in 1000 Genomes pilot project. These SNPs are placed in non-coding putative regulatory region (3’UTR and intronic regions respectively). Functional characterization of these SNPs can be seen in Table A in S1 File. The results suggest that these polymorphisms are placed in regulatory regions (TF binding sites as well as in sites of histone enhancer sites marks in several tissues), and alleles present different affinity for protein-DNA interaction (i.e. alternate C allele in both polymorphisms reduce the predicted affinity with proteins RXRA, SETDB1, Znf143, Myb when compared to wild allele T). The profile expression of interactive proteins (RXRA, SETDB1, Znf143, Myb) was evaluated in The Human Protein Atlas [11]. All the proteins showed a high expression level on the respiratory tissue, except Myb transcription factor, which presents a medium expression level. Taken together, our results suggest that rs113350588 and rs1048479 may alter the function of ST3GAL1 either directly through splicing regulation alteration and/or indirectly through LD with SNP with regulatory function.

There were no significant differences in the distribution of allele or genotype frequencies between patients (Table 3). A higher frequency of the GC and AT haplotypes was observed in patients who died (13.2% and 22.4%, respectively) when compared to patients who were not hospitalized (7.0% and 6.4%) and hospitalized patients who survived (4.1% and 8.1%) (Table 4). The influence of these haplotypes on the risk of more severe disease or death was evaluated using logistic regression models (Table 5). Patients carrying the GC haplotype did not exhibit a higher risk of more severe disease, but the risk of death due to infection with *Influenza A(H1N1)pdm09* was increased in this group (OR = 4.159, 95% CI = 1.55;11.12). The risk of severe disease and death was higher among patients carrying the AT haplotype (OR = 1.959, 95% CI = 1.09;3.52, and OR 2.856, 95% CI = 1.41;5.77, respectively).

**Table 3:** Allele and genotype frequency of the rs113350588 and rs1048479 polymorphisms of the *ST3GAL1* gene in patients infected with *Influenza A(H1N1)pdm09*. Genotypes and alleles are presented as number (%)

Polymorphism	Not hospitalized	Hospitalized		P
		Survived	Died	
rs1048479				
CC	42 (26.8)	24 (19.5)	14 (18.4)	
CT	73 (46.5)	54 (43.9)	34 (44.7)	0.282
TT	42 (26.8)	45 (36.6)	28 (36.8)	
C	157 (50.0)	102 (69.8)	62 (40.8)	0.065
T	157 (50.0)	144 (30.1)	90 (59.2)	
rs113350588				
AA	47 (29.9)	35 (28.5)	21 (27.6)	
AG	65 (41.1)	64 (52.0)	34 (44.7)	0.357
GG	45 (28.7)	24 (19.5)	21 (27.6)	
A	159 (50.6)	134 (54.5)	76 (50.0)	0.588
G	155 (49.4)	112 (45.5)	76 (50.0)	

**Table 4:** Haplotype frequency of the rs113350588 and rs1048479 polymorphisms of the *ST3GAL1* gene in patients infected with *Influenza A(H1N1)pdm09*. Haplotypes are presented as number (%)

Allele	Haplotype (rs113350588/rs1048479)	Not Hospitalized	Hospitalized		P
			Survived	Died	
1	AC	135 (43.0)	92 (37.4)	42 (27.6)	
2	GC	22 (7.0)	10 (4.1)	20 (13.2)	<0.001
3	AT	20 (6.4)	20 (8.1)	34 (22.4)	
4	GT	137 (43.6)	124 (50.4)	56 (36.8)	

**Table 5:** Association of haplotypes of the *ST3GAL1* gene with the risk of more severe disease or death in patients infected with *Influenza A(H1N1)pdm09*. The following covariates were included in the model: age, comorbidities, and European and African genetic ancestry. OR: odds ratio; 95% CI: 95% confidence interval.

Haplotypes	Hospitalization			Death		
	OR	95% CI	P	OR	95% CI	P
Reference^$^	—	—	—	—	—	—
GC carriers*	1.129	0.481;1.962	0.738	4.632	2.100;10.217	<0.001
AT carriers^#^	1.993	1.099;3.613	0.023	4.476	2.372;8.449	<0.001

^$^Reference: AC/AC, AC/GT, and GT/GT

*GC carriers: GC/AC, GC/GT, and GC/GC.

^#^AT carriers: AT/AC, AT/GT, and AT/AT.

## Discussion

On April 21^st^, 2009 the Centers for Disease Control and Prevention (CDC) reported two cases of infection with a new influenza virus strain which had occurred in California, USA [12]. This strain rapidly spread around the world and gave origin to the first influenza pandemic in the 21^st^ century [13]. In this pandemic, the fact that the incidence of severe disease was higher among young adults than among individuals older than 50 years called attention [14] and differed from observations made during annual epidemics caused by other human viral subtypes [15].

In the sample studied, the mean age of hospitalized patients was 22 years and the mean age of patients who died was 30 years. In August 2009, among the cases of pandemic influenza notified in 122 cities of the United States, more than 85% of confirmed deaths due to the pandemic strain occurred in individuals younger than 60 years, with a mean age at death of 37 years. In contrast, in epidemics caused by seasonal strains 90% of deaths occur in individuals older than 65 years and the estimated mean age at death is 76 years. The mean age at death caused by the pandemic strain is also lower than that observed in the influenza epidemics that occurred in 1957 and 1968 [16].

In the present study, two polymorphisms of the *ST3GAL1* gene, which encodes the Siaα2-3Galβ1- receptor, were investigated in patients with a diagnosis of influenza caused by the pandemic strain. Multivariate analysis demonstrated an association between the GC and AT haplotypes and severity or death due to the infection. Expression levels of sialyltransferase genes are known to differ according to tissue and type of cell, permitting regulation of the cellular pattern of sialylation and anticipating a complex specificity of these enzymes [17]. The enzyme encoded by *ST3GAL4* gene which transfers the sialic acid chain to a galactose residue, forming beta-galactoside alpha–2,3-sialyltransferase 4 (Galβ1-4GlcNAc), serves as a cell entry receptor of influenza H5N1. The expression patterns of this enzyme differ between tissues of the respiratory tract and also show interpersonal variability, influencing differences in the rate of infection with this virus in a population [18]. Similarly, higher expression of the Siaα2-6Gal(NAC) receptor was observed in the lung tissue of a young patient without comorbidities who died, when compared to three other patients who died and had important risk factors for severe infection with *Influenza A(H1N1)pdm09* [19].

Variability in the expression of the Siaα2-3Galβ1- receptor in tissues of the respiratory tract may also be related to variations in the manifestation of influenza caused by the 2009 pandemic strain. The rs113350588 and rs1048479 polymorphisms of the ST3GAL1 gene were predicted to play a role in the regulation and processing of transcription of this gene, influencing the availability of functional protein in the cell. The GC and AT haplotypes of the ST3GAL1 gene were more frequent in patients who died and determines a higher risk to this outcome. The presence of these haplotype variants should influence the expression or structure of Siaα2-3Galβ1- receptors in cells of the lower respiratory tract, facilitating entry of the virus into tissues and increasing viremia which, in turn, can lead to more severe presentation of the disease and can culminate in death. Functional studies would clarify the influence of these variants on enzyme expression and receptor formation.

The present study demonstrated for the first time the association between *ST3GAL1* gene haplotypes and the risk of more severe disease and death in patients infected with *Influenza A(H1N1)pdm09*. Studies of this gene in different world populations should help clarify the importance of these variants for the understanding of the role of host genetic variability in the clinical presentation and development of pandemic influenza.

## Material and Methods

### Study population

The study was divided into two phases: first, the presence of genetic variation in the *ST3GAL1* gene was evaluated in a small sample; second, the polymorphisms found were genotyped in a larger sample. Collection of the material during the two phases was accompanied by filling out a notification form of the Brazilian National System of Medical Care (SINAM) which contained the clinical data of the patient. The study was approved by the Research Ethics Committee of the Center of Tropical Medicine, Federal University of Pará, and all patients who agreed to the blood collection signed a free informed consent form. Underage participants (younger than 18 years n = 133) had the informed consents signed by parents to participate in the study. All informed consent forms were filed at Federal University of Pará.

### Selection of the polymorphisms

In a preliminary study, 201 blood and nasal aspirate and/or nasopharyngeal swab samples were collected from subjects of both genders and all age groups who had a clinical suspicion of flu syndrome caused by strain A(H1N1)pdm09 and who sought healthcare services in the metropolitan region of Belém, Pará, Brazil. Diagnostic confirmation of the strain was obtained at the Laboratory of Respiratory Viruses, Virology Section of the Evandro Chagas Institute (SEVIR/IEC), Ananindeua, Pará, using the SuperScript III ^TM^ One-step qRT-PCR System with Platinum Taq^®^ (Invitrogen Life Technologies^®^), according to the protocol recommended by the Centers for Disease Control and Prevention [20]. Genomic DNA was extracted from samples of 68 patients infected with *Influenza A(H1N1)pdm09* virus using the QIAamp DNA Mini Kit (Qiagen^®^) according to manufacturer instructions.

The primers for amplification of the six codifying regions of the *ST3GAL1* gene were designed using the Primer3 software [21] based on the reference sequence ENSG00000008513 (Table D in S1 File) [22]. After testing with the AutoDimer software, the primers were used in a polymerase chain reaction (PCR) to amplify each exon (numbered 4 to 9 according to the reference transcript ENST00000521180) in the 68 patients (Table D in S1 File) [22]. The amplicons were then sequenced using the Big Dye Terminator Kit (Applied Biosystem^®^) according to manufacturer specifications. The PCR conditions are described in Tables E and F in S1 File. Once obtained, the sequences were aligned at a similarity of at least 70% with 10 times refinement using the Geneious 5.5.6^®^ software to identify point mutations. The rs113350588 SNP in exon 4 and the rs1048479 SNP in exon 8 were detected in the sample studied, with minor allele frequencies of 50% and 40.4%, respectively (Table G in S1 File).

In order to evaluate the putative effect of both variants in ST3GAL1 regulation, *in sillico* analyses using HaploReg 3.0 [23] and Human Splicing Finder 3.0 [24] were performed to analyze the putative role regulatory function and on splicing activity respectively. HaploReg is a tool for exploring annotations of the noncoding genome at variants on haplotype blocks, draw on comprehensive data from the Encyclopedia of DNA Elements (ENCODE). Using LD information from the 1000 Genomes Project, genetic variants can be visualized along with their predicted chromatin state, their sequence conservation across mammals, and their effect on regulatory motifs[23]. Human Splicing Finder 3.0 tool [24], is a tool that helps studying the pre-mRNA splicing. It combines 12 different algorithms to identify and predict mutations’ effect on splicing motifs including the acceptor and donor splice sites, the branch point and auxiliary sequences known to either enhance or repress splicing. These algorithms are based on either PWM matrices, Maximum Entropy principle or Motif Comparison method.

### Genotyping of SNPs

In the second phase of the study, 356 patients were randomly selected among 1,524 cases of *Influenza A(H1N1)pdm09* from the northern and northeastern regions of Brazil, confirmed at the Evandro Chagas Institute. Diagnostic confirmation and DNA extraction were done as described for the first phase of the study. Allelic discrimination of the polymorphisms was performed in all samples by real-time PCR using the C_2771724_10 assay (rs1048479) and a custom assay (rs113350588) of the TaqMan^®^ system (Applied Biosystems^®^) according to manufacturer instructions.

### Population substructure

The proportions of African, European and Native American genetic ancestry in the 356 patients included in the second phase of the study were estimated using a panel of 48 ancestry informative markers as described elsewhere [25].

### Statistical analysis

Allele frequencies were estimated by direct counting. Hardy-Weinberg equilibrium was tested by chi-squared analysis. Haplotype frequencies and linkage disequilibrium were estimated with the Phase 2.1.1 software [26]. Differences in quantitative and qualitative characteristics between the groups of hospitalized patients, non-hospitalized patients and patients who died of the disease were verified by ANOVA, Fisher’s exact test and the Kruskal-Wallis test. Fisher’s exact test was also applied to analyze differences in allele frequencies of the haplotypes between the groups of patients. Logistic regression models were used to determine the association between *ST3GAL1* gene haplotypes and the severity of infection, adjusting for the following variables: age, European and African genetic ancestry and presence of comorbidities. All analyses were performed with the SPSS 18.0 software and a level of significance of p < 0.05 was adopted.

## Supporting Information

S1 File **Table A in S1 File. *In silico* functional analysis results for rs113350588 and rs1048479 and variants in Linkage disequilibrium**. *Refers to LD between the rs2142306, rs2736865 and rs1048479 polymorphism for 1000 Genome project. **Table B in S1 File. Frequency of the *ST3GAL1* gene haplotypes observed in patients infected with *Influenza A(H1N1)pdm09*. Table C in S1 File. *ST3GAL1* gene diplotypes frequencies observed in patients infected with *Influenza A(H1N1)pdm09*. Table D in S1 File. Sequence of the primers used for PCR amplification and nucleotide sequencing of the *ST3GAL1* gene. Table E in S1 File. Protocol for PCR amplification**. ^a^Mixture of deoxyribonucleotide triphosphates: dATP, dCTP, dGTP, and dTTP. ^b^A total of 35 cycles were performed for reactions with the exception of exon 8. ^c^Anneling: 65°C 2 cycles; 64°C 10 cycles; 62°C 10 cycles, and 60°C 15 cycles. **Table F in S1 File. Protocol for sequencing**. ^a^Performed with 35 cycles. **Table G in S1 File. Allele and genotype frequency of the *ST3GAL1* gene polymorphisms in 68 patients infected with *Influenza A(H1N1)pdm09***.
(DOCX)
