# Supplementary material for: Siaα2-3Galβ1- Receptor Genetic Variants Are Associated with Influenza A(H1N1)pdm09 Severity
Source: PLoS One. 2015 Oct 5;10(10):e0139681. doi: 10.1371/journal.pone.0139681 (PMC4593567; doi:10.1371/journal.pone.0139681)
Supplement: S1 File — Table A in S1 File. In silico functional analysis results for rs113350588 and rs1048479 and variants in Linkage disequilibrium. *Refers to LD between the rs2142306, rs2736865 and rs1048479 polymorphism for 1000 Genome project. Table B in S1 File. Frequency of the ST3GAL1 gene haplotypes observed in patients infected with Influenza A(H1N1)pdm09. Table C in S1 File. ST3GAL1 gene diplotypes frequencies observed in patients infected with Influenza A(H1N1)pdm09. Table D in S1 File. Sequence of the primers used for PCR amplification and nucleotide sequencing of the ST3GAL1 gene. Table E in S1 File. Protocol for PCR amplification. aMixture of deoxyribonucleotide triphosphates: dATP, dCTP, dGTP, and dTTP. bA total of 35 cycles were performed for reactions with the exception of exon 8. cAnneling: 65°C 2 cycles; 64°C 10 cycles; 62°C 10 cycles, and 60°C 15 cycles. Table F in S1 File. Protocol for sequencing. aPerformed with 35 cycles. Table G in S1 File. Allele and genotype frequency of the ST3GAL1 gene polymorphisms in 68 patients infected with Influenza A(H1N1)pdm09. (DOCX) [file pone.0139681.s001.docx]

**Table A. *In silico* functional analysis results for rs113350588 and rs1048479 and variants in Linkage disequilibrium**

| Variant | Ref | Alt | LD*  (r2; D')  AFR | LD*  (r2; D')  EUR | LD*  (r2; D')  AMR | LD*  (r2; D')  ASN | dbSNP  functional annotation | Splicing regulation | Regulatory motifs altered changed | Enhancer histone marks |
| --- | --- | --- | --- | --- | --- | --- | --- | --- | --- | --- |
| rs113350588 | G | A |  |  |  |  | synonymous (exon 4) | ESE broken (disruption of SF2/ASF sites) |  |  |
| rs1048479 | C | T |  |  |  |  | synonymous (exon 8) | New acceptor site |  |  |
| rs2142306 | T | C | 0.96; 0.98 | 0.65; 0.95 | 0.75; 0.93 | 0.99; 0.99 | 3'UTR |  | Myb,SETDB1 | LUNG, BLOOD, SKIN, LIVER, KIDNEY, PLACENT |
| rs2736865 | T | C | 0.97; 0.99 | 0.97; 1.0 | 0.98; 0.99 | 1.0; 1.0 | intron |  | RXRA, SETDB1,  Znf143 |  |

*Refers to LD between the rs2142306, rs2736865 and rs1048479 polymorphism for 1000 Genome project

**Table B. Frequency of the *ST3GAL1* gene haplotypes observed in patients infected with *Influenza A(H1N1)pdm09.***

| **Alleles** | **Haplotypes** | | **N** | **Frequency (%)** |
| --- | --- | --- | --- | --- |
|  | **rs113350588** | **rs1048479** |  |  |
| 1 | A | C | 269 | 37.8 |
| 2 | G | C | 52 | 7.3 |
| 3 | A | T | 74 | 10.4 |
| 4 | G | T | 317 | 44.5 |

**Table C. *ST3GAL1* gene diplotypes frequencies observed in patients infected with *Influenza A(H1N1)pdm09.***

| Diplotypes | N | Frequency (%) |
| --- | --- | --- |
| AC/AC | 59 | 16.6 |
| AC/GC | 11 | 3.1 |
| AC/AT | 24 | 6.7 |
| AC/GT | 116 | 32.6 |
| GC/GC | 10 | 2.8 |
| GC/GT | 21 | 5.9 |
| AT/AT | 7 | 2.0 |
| AT/GT | 36 | 10.1 |
| GT/GT | 72 | 20.2 |

**Table D. Sequence of the primers used for PCR amplification and nucleotide sequencing of the *ST3GAL1* gene.**

| Gene Location | Sequence (5’- 3’) | Amplicon (pb) | Coding region (pb) |
| --- | --- | --- | --- |
| Exon 4 | *F_*CACTCTTATCCAGATCCCCACT | 488 | 306 |
|  | *R_*ATGGAAGAAAAATCCCAGAGGT |  |  |
| Exon 5 | *F_*GTCCCTGGGTAAGTTCAGTCC | 496 | 197 |
|  | *R_*GACACCTCCACTTCCTCCTTC |  |  |
| Exon 6 | *F_*GGACACTAAAGCTTGTGGTTTGT | 488 | 180 |
|  | *R_*GTGAGCCTCCAGTGTGACTTC |  |  |
| Exon 7 | *F_*CAATGCCGTACCTTAGAGCA | 392 | 46 |
|  | *R_*AAAGGTGAGGAGGACTTGCTC |  |  |
| Exon 8 | *F_*TGTGCCAGAACTATTGCTCCT | 509 | 120 |
|  | *R_*GGAAAGATGGACACTCATCCA |  |  |
| Exon 9 | *F_*CCACTCAAGACAGGTTCCAAG | 466 | 174 |
|  | *R_*CACCTCTGAGAAAGGAAGCCT |  |  |

**Table E. Protocol for PCR amplification.**

|  |  | **exon 9** | **exon 8** | **exon 7** | **exon 6** | **exon 5** | **exon 4** |
| --- | --- | --- | --- | --- | --- | --- | --- |
| **PCR reagents *(µL)*** |  |  |  |  |  |  |  |
| H_2_O DNAase/RNAase free |  | 17.55 | 16.3 | 17.3 | 17.55 | 16.55 | 17.55 |
| dNTPs^a^ |  | 2 | 2 | 2 | 2 | 2 | 2 |
| 10X Reaction Buffer |  | 2.5 | 2.5 | 2.5 | 2.5 | 2.5 | 2.5 |
| MgCl_2_ |  | 0.75 | 1 | 1 | 0.75 | 0.75 | 0.75 |
| Primer ( Forward ) |  | 0.5 | 1 | 0.5 | 0.5 | 1 | 0.5 |
| Primer ( Reverse ) |  | 0.5 | 1 | 0.5 | 0.5 | 1 | 0.5 |
| DNA (20ng) |  | 1 | 1 | 1 | 1 | 1 | 1 |
| Taq DNA Polimerase |  | 0.2 | 0.2 | 0.2 | 0.2 | 0.2 | 0.2 |
| **Thermocycling conditions^b^** |  |  |  |  |  |  |  |
| ***Temperature (°C )*** |  |  |  |  |  |  |  |
| Initial denaturation |  | 95 | 95 | 95 | 95 | 95 | 95 |
| Denaturation |  | 95 | 95 | 95 | 95 | 95 | 95 |
| Annealing |  | 61 | 65-60^c^ | 60 | 61 | 61 | 60 |
| Extension |  | 72 | 72 | 72 | 72 | 72 | 72 |
| Final extension |  | 72 | 72 | 72 | 72 | 72 | 72 |
| Cooling |  | 5 | 5 | 5 | 5 | 5 | 5 |
| **Time (min)** |  |  |  |  |  |  |  |
| Initial denaturation |  | 5 | 5 | 5 | 5 | 5 | 5 |
| Denaturation |  | 0.6 | 0.6 | 0.6 | 0.6 | 0.6 | 0.6 |
| Annealing |  | 1 | 1 | 1 | 1 | 1 | 1 |
| Extension |  | 1 | 1.3 | 1 | 1.3 | 1 | 1.3 |
| Final extension |  | 30 | 40 | 30 | 40 | 30 | 40 |
| Cooling |  | 5 | 5 | 5 | 5 | 5 | 5 |

^a^Mixture of deoxyribonucleotide triphosphates: dATP, dCTP, dGTP, and dTTP.

^b^A total of 35 cycles were performed for reactions with the exception of exon 8.

^c^Anneling: 65 °C 2 cycles; 64 °C 10 cycles; 62 °C 10 cycles, and 60 °C 15 cycles.

**Table F. Protocol for sequencing.**

|  |  | **exon 9** | **exon 8** | **exon 7** | **exon 6** | **exon 5** | **exon 4** |
| --- | --- | --- | --- | --- | --- | --- | --- |
| **PCR reagents *(µL)*** |  |  |  |  |  |  |  |
| H_2_O DNAase / RNAase free |  | 15.25 | 15 | 15.25 | 15 | 15.25 | 15 |
| Big Dye v. 3.1 |  | 0.5 | 0.5 | 0.5 | 0.5 | 0.5 | 0.5 |
| Big Dye buffer |  | 3 | 3 | 3 | 3 | 3 | 3 |
| Primer (Forward ) |  | 0.25 | 0.5 | 0.25 | 0.5 | 0.25 | 0.5 |
| Amplicon |  | 1 | 1 | 1 | 1 | 1 | 1 |
| **Thermocycling conditions^a^** |  |  |  |  |  |  |  |
| ***Temperature (°C )*** |  |  |  |  |  |  |  |
| Initial denaturation |  | 95 | 95 | 95 | 95 | 95 | 95 |
| Denaturation |  | 95 | 95 | 95 | 95 | 95 | 95 |
| Annealing |  | 61 | 60 | 60 | 60 | 60 | 60 |
| Extension |  | 72 | 72 | 72 | 72 | 72 | 72 |
| Final extension |  | 5 | 5 | 5 | 5 | 5 | 5 |
| **Time (min)** |  |  |  |  |  |  |  |
| Initial denaturation |  | 5 | 5 | 5 | 5 | 5 | 5 |
| Denaturation |  | 0.6 | 0.6 | 0.6 | 0.6 | 0.6 | 0.6 |
| Annealing |  | 1 | 1 | 1 | 1 | 1 | 1 |
| Extension |  | 1 | 1.3 | 1 | 1.3 | 1 | 1.3 |
| Final extension |  | 5 | 5 | 5 | 5 | 5 | 5 |

^a^Performed with 35 cycles.

**Table G. Allele and genotype frequency of the *ST3GAL1* gene polymorphisms in 68 patients infected with *Influenza A(H1N1)pdm09*.**

| Variant | N | Frequency (%) |
| --- | --- | --- |
| **rs113350588** |  |  |
| AA | 17 | 25.0 |
| AG | 34 | 50.0 |
| GG | 17 | 25.0 |
| A | 68 | 50.0 |
| G | 68 | 50.0 |
| **rs1048479** |  |  |
| CC | 13 | 19.2 |
| CT | 29 | 42.6 |
| TT | 26 | 38.2 |
| C | 55 | 40.4 |
| T | 81 | 59.6 |
